# WSPMaker: a web tool for calculating selection pressure in proteins and domains using window-sliding

**DOI:** 10.1186/1471-2105-9-S12-S13

**Published:** 2008-12-12

**Authors:** Yong Seok Lee, Tae-Hyung Kim, Tae-Wook Kang, Won-Hyong Chung, Gwang-Sik Shin

**Affiliations:** 1 Korean Bioinformation Center, KRIBB, Daejeon 305-806, Korea; 2 Medical Genomics Research Center, KRIBB, 52 Eoeun-dong, Yuseong-gu, Daejeon 305-806, Republic of Korea; 3 Department of Biology, Kyungpook National University, Taegu 702-701, Korea; 4 Department of Computer Engineering, Kyungpook National University, Taegu 702-701, Korea

## Abstract

**Background:**

In the study of adaptive evolution, it is important to detect the protein coding sites where natural selection is acting. In general, the ratio of the rate of non-synonymous substitutions (Ka) to the rate of synonymous substitutions (Ks) is used to estimate either negative or positive selection for an entire gene region of interest. However, since each amino acid in a region has a different function and structure, the type and strength of natural selection may be different for each amino acid. Specifically, domain sites on the protein are indicative of structurally and functionally important sites. Therefore, Window-sliding tools can be used to detect evolutionary forces acting on mutation sites.

**Results:**

This paper reports the development of a web-based tool, WSPMaker (Window-sliding Selection pressure Plot Maker), for calculating selection pressures (estimated by Ka/Ks) in the sub-regions of two protein-coding DNA sequences (CDSs). The program uses a sliding window on DNA with a user-defined window length. This enables the investigation of adaptive protein evolution and shows selective constraints of the overall/specific region(s) of two orthologous gene-coding DNA sequences. The method accommodates various evolutionary models and options such as the sliding window size. WSPmaker uses domain information from Pfam HMM models to detect highly conserved residues within orthologous proteins.

**Conclusion:**

WSPMaker is a web tool for scanning and calculating selection pressures (estimated by Ka/Ks) in sub-regions of two protein-coding DNA sequences (CDSs).

## Background

In researching adaptive evolution processes, it is important to detect protein coding genes that are under an active natural selection process. Traditionally, this is described as the ratio of Ka/Ks. Ka is the rate of non-synonymous substitution and Ks is the rate of synonymous substitution. The Ka/Ks ratio can be used to estimate either negative or positive selection tendencies for genes of interest [1-3]. However, most Ka/Ks ratio values of complete coding genes have been found to be too low to be easily detected. This is because, in general, non-synonymous substitutions occur much less frequently than synonymous substitutions [4]. Therefore, regions that may have important protein function and structure showing Ka/Ks values over 1 are not readily detectable with classical methods. Conventional methods often use sliding windows for detecting high Ka/Ks values that may represent high selection pressure at a single amino acid site or gene region [5,6]. However, these methods can be ineffective at detecting good candidate sites when natural selection is under way or when a small number of alignable sequences are provided.

Recently, the Selecton program [7] has been developed to detect the evolutionary effects on a single amino acid site within the three-dimensional (3D) protein structure. This tool is powerful at detecting active substitution regions if the 3D structures of genes are available and if at least five homologous sequences are provided. Unfortunately, not all genes have corresponding 3D structures, and it may be difficult to provide five homologous sequences from five different species. This paper presents WSPMaker (Window-Sliding Selection Pressure plot maker), a new web tool for assisting in the detection of gene regions under differential selection pressure when only two pair-aligned protein-coding sequences are provided.

The most important feature of the WSPMaker is that it compares the selection constraints of domain and non-domain regions. It provides a simple (or effective) method to detect for defining positive and negative selection pressure regions within protein-coding sequences.

## Results

**Figure 1:**
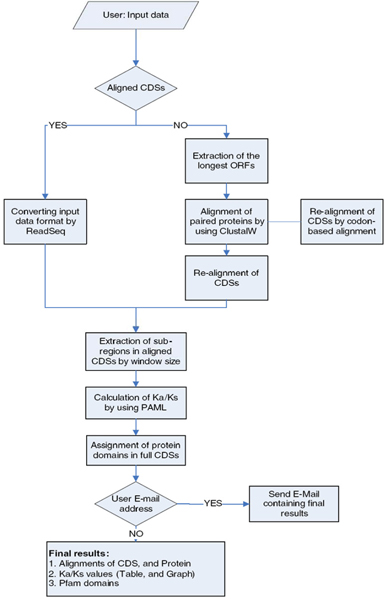
A schematic overview of the WSPMaker.

WSPMaker (Window-sliding Selection pressure Plot Maker) uses the ClustalW sequence alignment tool [8]. As a default, it requires two orthologous mRNA sequences. It translates them into protein sequences and chooses the longest open reading frame (ORF) by a user-selected genetic code table. This step is skipped if users submit their own alignment of orthologous CDSs. This alignment file is extracted to window-sliding sub-regions with the window size provided by the users. The users may select an option for assigning functional domain regions in the proteins. WSPMaker is able to produce a plotted graph with Ka/Ks value for different regions of the coding gene, and emphasizes specific regions of proteins that are under selection pressure, with five different user-defined methods to detect selection and selection values (positive and negative values). The schematic overview and the data flow of WSPMaker are shown in Figure 1.

### Implemented evolutionary models

WSPMaker implements several different evolutionary models widely used to estimate the level of selection operating on a gene or coding region. In the 'Single Size' option, there are three model pairs (M1a v. M2a: nearly neutral v. positive selection, M7 v. M8: beta v. beta & w, and M0 v. M3: one-ratio v. discrete). The first pair includes M1a (nearly neutral) and M2a (positive selection). The second pair includes M7 (beta) and M8 (beta & ω), while the third pair includes M0 (one ratio) and M3 (discrete). Comparison of these model pairs allows the for statistical testing of the hypothesis that there is positive selection on proteins (alternative hypothesis model) against the null hypothesis model. The output is the likelihood of each model, allowing for a comparison using a likelihood ratio test (LRT). The program predicts when a site is undergoing positive selection. In the second option, 'User Defined Size,' there are two models. The Yang and Nielsen model [9] and the Nei and Gojobori [2] model are commonly used in estimating synonymous and non-synonymous substitution rates and detecting positive selection in protein-coding sequences.

### Input data format and parameters

WSPMaker takes a default input data type of user-curated orthologous DNA coding sequence pairs. WSPMaker allows users to submit various sequence formats, such as FASTA, EMBL, GCG, ClustalW, and Phylip/Phylip4, for the input data containing codon-based alignment of paired sequences. WSPMaker provides a choice of one of the 17 NCBI genetic codes [10] for automatically generated codon-based alignment after translating orthologous CDSs. There are two additional advanced options: 'Single Size' and 'User Defined Size.' If users select 'Single Size,' they can choose one of three models to detect the sites that are under significant selection pressure. The default window and sliding size is 3 bp for calculating the Ka/Ks ratio. The 'User Defined Size' parameter is for calculating Ka/Ks using a user-defined sliding window size, and allows the users to choose one of two models in common use (Yang and Nielsen & Nei and Gojobori models). The default window size is 6% of the given gene sequence. This value is set empirically and can be altered. In addition, users can set positive or negative threshold values (defaults are one). Hmmpfam 2.3.2 was used to analyze domain distribution among all orthologous genes and to extract pfam domain sequences [11]. A threshold E-value <= 0.5 gave the best quality results, and thus was used in the present system. This domain architecture of the input orthologous genes was visualized with functional domains using pfam in the resulting web interface.

### Output

**Figure 2:**
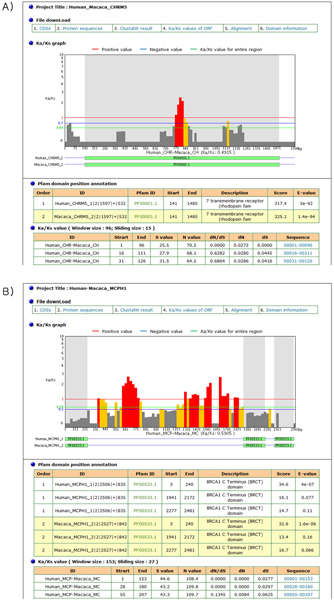
An example of the WSPMaker main output page. The calculated Ka/Ks ratios are plotted as slim bars within a default sliding window size. A) WSPMaker results for MCPH1. B) WSPMaker results for CHRM5.

The results page consists of four sections. They are 1) file download, 2) Ka/Ks graph, 3) Pfam domain position annotation, and 4) Ka/Ks calculation. An example output is shown in Figure 2. The first section contains the files available to download, the input and intermediate results produced in the automatic pipeline of the WSPMaker server. "CDSs" provides the longest DNA sequence with a start codon. "Protein sequence" provides the translated amino acid sequences. "ClustalW result" provides the aligned pairwise sequences using the ClustalW program. "Ka/Ks values of ORF" provides the Ka/Ks ratio generated by the codeml program in the PAML package [12]. "Pfam Annotation" provides the information on the Pfam domains. The second section contains a Ka/Ks graph, which shows the window-sliding selection pressure between two orthologous coding genes that have been entered in the input page. Each sliding window's Ka/Ks value can be displayed as a colored bar; a red bar means that the Ka/Ks value of its position is equal to or greater than the positive selection value (drawn as a horizontal red line on the graph), and therefore that the region has undergone positive selection. A yellow bar means that the score is equal to or greater than the negative selection value (drawn as a horizontal blue line) and lower than the positive selection value, and therefore that this region has undergone neutral evolution. A dark-gray bar means that the score is lower than the negative value, indicating a purely negative selection. The bars that have Ka/Ks values over 10 have been treated as 0-scored bars due to alignment errors or gaps. To highlight the the region of lower Ka/Ks values, the bar heights have been scaled in a logarithmic manner. Optionally, there is one more section ('Likelihood ratio test of positive selection') in the 'Single Size' option. This section compares the significance levels of two given models by performing the likelihood comparison (LRT test). The green-colored domain box has been positioned below the Ka/Ks graph, and the matched area has been highlighted by the light-gray color in the graph. When the mouse points to a bar, a tool-tip is shown that contains the selected bar's information in the Ka/Ks calculation. When the mouse points to a domain box, a tool-tip is shown that contains the selected domain's info in the Pfam Domain Position. The third section is the Pfam Domain Position Annotation table, which contains the analyzed information of each domain. The last section shows the Ka/Ks values, which contains the analyzed information of each sliding window.

## Applying WSPMaker to biological cases

Here, we give an example illustrating WSPMaker's use for detecting positive selection (Figure 2).

**Figure 3:**
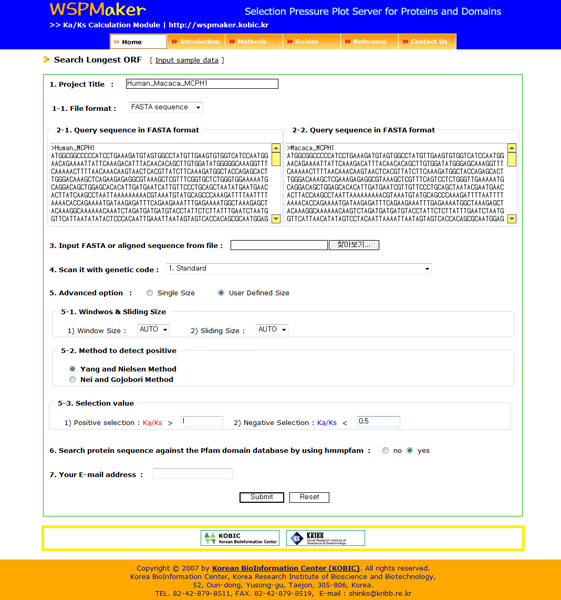
An example of the WSPMaker main input page. Users can paste in or upload sequences to calculate the Ka/Ks values of sub-regions in a CDS.

The subset of genes implicated in nervous system development is known to display significantly higher rates of protein evolution in primates than in rodents [13]. The higher average Ka/Ks values of nervous system genes in primates are suggestive of adaptive evolution, especially a positive selection process. However, none of the genes have Ka/Ks ratios greater than one with previous tests of adaptive evolution. Thus, to detect interesting evolutionary signatures, a good statistical power and other test methods are necessary. One such method is our MSPMaker, which scans and calculates selection pressure (estimated by Ka/Ks) in sub-regions of two protein-coding DNA sequences (CDSs). Here, nervous system genes MCPH1 and CHRM5 were used as inputs for the WSPMaker server. Disturbing the MCPH1 gene can result in a severe reduction in brain size (microcephaly), and CHRM5 is related to defects in acquiring reward-mediated behaviour. Both genes showed Ka/Ks values lower than one in primates (0.833 for MCPH1 and 0.242 for CHRM5) with the traditional methods, despite the fact that phenotypes of the nervous system such as brain size had apparently undergone far greater selection pressure in primates than in most other mammals. To test selection pressure in the sub-regions, we ran WSPMaker with human and macaque sequences of each gene. Options were set as follows: 'User Defined Size,' the default widow size and sliding size, and the Yang and Nielsen method. The results of the run are shown in Figure 3. The outputs showed many gray-colored regions, indicating that these regions have undergone neutral evolution, as was shown in traditional methods. However, there were exceptions in some regions, where the red colors indicated Ka/Ks values higher than one. We suggest that these regions have undergone amino acid fixations during primate evolution, even though the entire genes followed neutral evolution. In addition, the domain information provided by WSPMaker lets users compare the selection pressure on the protein domain regions and understand which particular positions are functionally important.

## Conclusion

WSPMaker is a useful web-based visualization tool that shows which DNA sub-regions of coding sequences are under strong selection pressure. It can assist researchers in easily detecting candidate genes and partial gene regions for fast base substitution in functional and evolutionary analyses, using its window-sliding visual graphs. The WSPMaker server can be accessed at .

## Competing interests

The authors declare that they have no competing interests.

## Authors' contributions

YSL, THK, and TWK wrote the code for WSPMaker. WHC worked on the visual display of the results. GSS designed the overall web site and coordinated the project. YSL wrote the main draft of the paper. All authors have read and approved the final manuscript.
